# High-Dose Aspirin Is Associated with Anemia and Does Not Confer Benefit to Disease Outcomes in Kawasaki Disease

**DOI:** 10.1371/journal.pone.0144603

**Published:** 2015-12-10

**Authors:** Ho-Chang Kuo, Mao-Hung Lo, Kai-Sheng Hsieh, Mindy Ming-Huey Guo, Ying-Hsien Huang

**Affiliations:** 1 Department of Pediatrics, Kaohsiung Chang Gung Memorial Hospital and Chang Gung University College of Medicine, Kaohsiung, Taiwan, 83301; 2 Kawasaki Disease Center, Kaohsiung Chang Gung Memorial Hospital, Kaohsiung, Taiwan, 83301; The University of Hong Kong, HONG KONG

## Abstract

**Background:**

Kawasaki disease (KD) is also known as multiple mucocutaneous lymph node syndrome of systemic vasculitis and is a leading cause of coronary artery lesions (CAL) in childhood. Intravenous immunoglobulin (IVIG) has been proven to effectively reduce the incidence of CAL, but the role and effect dose of aspirin in KD is still unclear. Moreover, overt bleeding and anemia are associated with the use of aspirin, and anemia is common in patients with KD. Thus, the aim of this study was conducted to compare the treatment efficacy, degree of anemia and inflammation, and changes in serum hepcidin in children who received a combination of high-dose aspirin and IVIG in the acute stage of KD, and those who received IVIG alone.

**Materials and Methods:**

KD patients from two medical centers were retrospectively analyzed from 1999–2009. All patients were initially treated with a single dose of IVIG (2 g/kg) as the standard care of treatment. In group 1, high-dose aspirin was prescribed (> 30 mg/kg/day) until the fever subsided, and then low-dose aspirin (3–5 mg/kg/day) was prescribed until all the inflammation signs had resolved. In group 2, low-dose aspirin was prescribed without high-dose aspirin. Laboratory data were collected for analysis in both groups.

**Results:**

A total of 851 KD patients (group 1, N = 305, group 2, N = 546) were enrolled in this study. There were no significant differences between group 1 and group 2 in terms of gender (p = 0.51), IVIG resistance rate (31/305 vs. 38/546, p = 0.07), CAL formation (52/305 vs. 84/546, p = 0.67), and duration of hospitalization (6.3 ± 0.2 vs. 6.7 ± 0.2 days, p = 0.13). There were also initially no significant differences in total white blood cell count, hemoglobin level, platelet count, and CRP before IVIG treatment between groups (all p>0.1). After IVIG treatment, group 1 had significantly lower levels of hemoglobin (p = 0.006) and higher CRP (p<0.001) as well as a smaller decrease in CRP level (p = 0.012). Furthermore, there was also a higher serum level of hepcidin and a delayed decrease in hepcidin level after receiving IVIG in group 1 (p = 0.04 and 0.02, respectively).

**Conclusions:**

These results provide evidence demonstrating that high-dose aspirin in the acute phase of KD **does not** confer any benefit with regards to inflammation and it does not appear to improve treatment outcomes. Therefore, high-dose aspirin is unnecessary in acute phase KD.

## Introduction

Kawasaki disease (KD) is known as a multiple mucocutaneous lymph node syndrome and an acute febrile systemic vasculitis. Although it was described by Kawasaki et al. in 1974,[1] its exact etiology is still unknown. KD occurs most commonly in children, particularly those under 2 years of age. The clinical presentations of KD are prolonged fever for more than five days, bulbar conjunctivitis, oral mucosal inflammation, polymorphous skin rashes, erythema and peeling of the tips of the hands and feet, and non-suppurative lymphadenopathy.[2, 3] In developed countries, KD is the most common acquired heart disease in children,[2–4] and its most serious complication are coronary artery lesions (CAL), which have been reported in 20–25% of untreated children.[5, 6]

Aspirin has been administered in KD treatment in the past decades, even before the treatment of intravenous immunoglobulin (IVIG).[5] It is conventionally believed that the combination of high-dose aspirin and IVIG may have additive anti-inflammatory, anti-pyretic, and anti-platelet effects. A multicenter study group reported that a single high dose of 2 g/kg IVIG plus aspirin could lower the incidence of aneurysms to 3–5% and also shorten the duration of fever.[6, 7]. Higher dose of aspirin (80–100 mg/kg per day) is most used during the acute febrile state of KD in North America,[8] though in Japan, concerns about hepatic toxicity have led to a reduced moderate dose (30–50 mg/kg per day) of aspirin.[9]. As there is no standard aspirin dose for KD, many centers suggest a lower one as soon as the patient gets afebrile. A further aspirin dose reduction to 3–5 mg/kg/day is then advised, usually by 6–8 weeks after the disease onset. If the children develop CAL, low-dose aspirin (or other anti-platelets) is continued until the echocardiographic findings are within normal range.

However, the use of high-dose aspirin in the acute febrile stage of KD i may have adverse effects, such as overt bleeding and anemia.[10] Recently, Lee et al. reported that high-dose aspirin shortens the duration of fever, and that treatment without aspirin in the acute phase has no influence on the response to IVIG, resolution of inflammation, or the development of CAL.[11] Moreover, Hsieh et al. reported a case series of 162 KD patients in whom treatment without high-dose aspirin in the acute febrile stage of KD had no benefit on the duration of fever or the incidence of CAL.[12] Aspirin has also been documented to have a possible hemolytic effect in individuals with glucose-6-phosphate dehydrogenase (G6PD) deficiency.[13] In children with G6PD deficiency who develop KD, IVIG alone may be a better alternative. Chen el al.[10] reported a child with G6PD-deficiency and KD for whom aspirin was not prescribed, and the fever subsided after single high-dose IVIG treatment with a good disease outcome (neither IVIG resistance nor CAL formation). In addition, anemia is a common finding in KD patients, and it is related to a more prolonged duration of fever.[14] Although we previously found that hemoglobin levels were significantly decreased in KD after IVIG treatment, it is still unknown whether high-dose aspirin exacerbates anemia in KD patients.[15] This study, conducted in two medical centers in Taiwan, aimed to compare the treatment efficacy, degree of anemia and inflammation, and changes in serum hepcidin among children who received a combination of high-dose aspirin and IVIG in the acute stage of KD, and those who received IVIG alone.

## Patients and Methods

### Patients

A total of 851 KD patients were enrolled in this retrospective study. All patients were children who fulfilled the criteria for KD [16] and had been treated with IVIG on the first day of diagnosis of KD at Kaohsiung Chang Gung Hospital or Veterans General Hospital-Kaohsiung, Taiwan. This is a retrospective study in which all data were collected from medical charts after patients had received standard-of-care treatment. The authors were not involved in allocating patients to group 1 and group 2.This study was approved by the Institutional Review Board of the Chang Gung Memorial Hospital and Veterans General Hospital-Kaohsiung. Written informed consent had been obtained from the guardians for serum hepcidin assay. The choice of high-dose aspirin is dependent on different doctors' opinions. Patients in group 1 received IVIG along with high-dose aspirin (> 30 mg/kg/day) and were started on low-dose aspirin (3–5 mg/kg/day) as a single daily dose after fever subsided and until all signs of inflammation resolved. In contrast, patients in group 2 received IVIG alone in the acute phase, and after the fever subsided were started on low-dose aspirin (3–5 mg/kg/day) as a single daily dose, which was continued until all signs of inflammation had resolved. As described in detail previously [12, 15, 17], laboratory data including complete blood count, C-reactive protein (CRP), hemoglobin level, duration of hospitalization, IVIG treatment response, and CAL formation rate were collected for analysis in both groups. CAL was defined as an internal diameter of at least 3 mm of the coronary artery (4 mm if the subject was over the age of 5 years), or an internal diameter of a segment at least 1.5 times as large as that of an adjacent segment in echocardiography.[14, 18] IVIG responsiveness was defined as the abatement of fever within 48 h of completing IVIG treatment with no recurrence (temperature >38°C) for at least seven days accompanied by marked improvement or normalization of inflammatory signs. [4, 19, 20]Peripheral blood was examined before IVIG treatment (within 24 hours), and within 3 days after completing the initial IVIG treatment. White blood cell count, CRP level and hemoglobin level were determined as part of standard hospital care and for further analysis between the groups.

The plasma level of hepcidin was measured in 80 of the patients. Blood samples were immediately placed in heparin-containing tubes, and the remaining aliquots of plasma were stored at -80°C until the assay.

### Laboratory measurements

#### Measurement of cytokines by enzyme-linked immunoassay (ELISA)

The ELISA we used for hepcidin-25 amino acid is a commercially available and competitive assay using synthetic hepcidin (Catalog Number: S-1337, Bachem Biosciences, St. Helens, United Kingdom, range: 0–25 ng/ml) for standardization, and the methodology and performance characteristics have been previously described.[15]

### Statistical analysis

All data are presented as mean ± standard error. Quantitative data were analyzed using Student’s t test, and changes in the data before and after IVIG treatment were tested using the paired sample *t-*test. Two-sided p values of less than 0.05 were considered statistically significant. All statistical tests were performed using SPSS version 14.0 for Windows XP (SPSS, Inc., Chicago, USA).

## Results

### High-dose aspirin in the acute stage of KD did not benefit the rates of CAL and IVIG resistance

851 KD patients (group 1, N = 305, group 2, N = 546) were enrolled in this study (Table 1). There were no significant differences between group 1 and group 2 in gender distribution (p = 0.51), IVIG resistance (31/305 vs. 38/546, p = 0.07), incidence of CAL formation (52/305 vs. 84/546, p = 0.67), and hospitalization days (6.3 ± 0.2 vs. 6.7 ± 0.2, p = 0.13). The groups also showed no difference in total white blood cell count (p = 0.99), hemoglobin level (p = 0.11), platelet count (p = 0.65) and CRP level (p = 0.99) before IVIG treatment (Table 2).

**Table 1:** Characteristics of the 851 Kawasaki disease patients.

	Group 1 (N = 305)	Group 2 (N = 546)	P value
Male gender (%)	201 (65.9)	338 (61.9)	0.51
IVIG resistance (%)	31 (10.2)	38 (7.0)	0.07
CAL formation (%)	52 (17.0)	84 (15.4)	0.67
Total hospital days	6.3 ± 0.2	6.7 ± 0.2	0.13

Group 1: patients with Kawasaki disease treated with high dose aspirin (>30mg/kg/day) in the acute stage. Group 2: Patients with Kawasaki disease treated without high dose aspirin (>30mg/kg/day) in the acute stage. IVIG: intravenous immunoglobulin; CAL: coronary artery lesions

**Table 2:** Laboratory data before IVIG treatment in the Kawasaki disease patients.

	Group 1 (N = 305)	Group 2 (N = 546)	P value
Total white blood count (/mm^3^)	13694 ± 375.9	13700 ± 427.9	0.99
Hemoglobin (g/dL)	10.81 ± 0.08	10.97 ± 0.06	0.11
Platelets (10^4^/mm^3^)	34.2 ± 0.9	34.8 ± 1.1	0.65
CRP (mg/L)	98.9 ± 4.9	92.1 ± 4.3	0.30

IVIG: intravenous immunoglobulin; CRP: C reactive peptide

### High-dose aspirin affected hemoglobin levels and delayed the decrease of CRP levels after IVIG therapy

As shown in Tables 2 and 3, the KD patients had significantly lower hemoglobin levels after IVIG treatment (p<0.001 in both group 1 and group 2), which is consistent with our previous findings.[14, 15] Moreover, there were significantly lower hemoglobin levels in group 1 than in group 2 after IVIG treatment (10.42 ± 0.08 vs. 10.70 ± 0.07 g/dl, p = 0.006) (Table 3). An elevated CRP level is almost always found in patients with KD, which then decreases after IVIG treatment.[6] In addition, a lower fractional change in CRP may be a useful and important marker to predict initial IVIG resistance and CAL in KD patients.[11, 14] As shown in Table 3, we found a significantly higher CRP level (54.7 ± 4.4 vs. 35.9 ± 2.8 mg/l, p<0.001) and a delayed decrease in the level of CRP (42.0 ± 5.0 vs. 58.7 ± 4.2 mg/dl, p = 0.012) in group 1 compared to group 2 after IVIG treatment.

**Table 3:** Laboratory data after IVIG treatment in the Kawasaki disease patients.

	Group 1 (N = 305)	Group 2 (N = 546)	P value
Total leukocytes (/mm^3^)	9803 ± 345.9	9734 ± 393.1	0.89
Hemoglobin (g/dL)	10.42 ± 0.08	10.70 ± 0.07	0.006**
Platelets (10^4^/mm^3^)	45.9 ± 1.9	43.8 ± 1.5	0.30
CRP (mg/L)	54.7 ± 4.4	35.9 ± 2.8	<0.001**
Decrease in CRP	42.0 ± 5.0	58.7 ± 4.2	0.012*

IVIG: intravenous immunoglobulin; CRP: C reactive peptide;

* p<0.05;

**p<0.005

### A delayed decrease in plasma hepcidin levels was associated with high-dose aspirin usage

We previously showed that anemia in patients with KD is related to markedly increased hepcidin expression which results in functional iron deficiency. Moreover, the serum hepcidin levels are significantly decreased after IVIG treatment.[15] For these reasons, we investigated the association between high-dose aspirin and serum hepcidin levels in the 80 KD patients with available data. Before IVIG treatment, there was no significant difference in hepcidin levels between group 1 and group 2 (220.89 ± 36.84 vs. 243.52 ± 26.97 ng/ml, p = 0.64) (Table 4); after IVIG treatment, there was only a significant decrease in hepcidin level in group 2 (p<0.001) but not in group 1 (p = 0.19). In addition, the hepcidin level was significantly higher (163.98 ± 52.94 vs. 81.48 ± 13.56 ng/ml, p = 0.04) and the change in hepcidin level significantly lower (56.90 ± 46.42 vs. 162.04 ± 22.66 ng/ml, p = 0.02) in group 1 than in group 2. This suggests that high-dose aspirin influenced the decrease in hepcidin expression after IVIG treatment.

**Table 4:** Hepcidin levels in the Kawasaki disease patients.

	Group 1 (N = 25)	Group 2 (N = 55)	P value
Pre-IVIG (ng/ ml)	220.89 ±36.84	243.52 ± 26.97	0.64
Post-IVG (ng/ ml)	163.98 ± 52.94	81.48 ± 13.56	0.04*
Decrease level (ng/ ml)	56.90 ± 46.42	162.04 ± 22.66	0.02*

IVIG: intravenous immunoglobulin, pre-IVIG, 24 hours before IVIG treatment; post-IVIG, within 3 days after IVIG treatment.

*p<0.05.

## Discussion

Our results reveal that high-dose aspirin in the acute phase of KD does confer any benefit to disease outcomes; furthermore, it may be harmful with regards to inflammation. In addition, this is the first study to demonstrate that high-dose aspirin markedly impairs the decrease in hepcidin level and may be associated with a decrease in hemoglobin in the acute phase of KD. Anemia is a common finding in KD, and in our previous study, we provided a mechanism to explain anemia in KD patients: it is related to a markedly increased hepcidin expression.[15] Moreover, the current study also introduces the novel observation that the lower decrease in hepcidin level after high-dose aspirin treatment was associated with a significantly lower hemoglobin level after IVIG treatment in the KD patients.

The use of aspirin in the treatment of KD has been recommended for several decades.[21] It was shown that high-dose aspirin has anti-inflammatory and low-dose has anti-platelet activity. However, it does not appear to lower the frequency of the development of coronary abnormalities.[11, 22] Saulsbury et al. first reported comparisons of two dosages of aspirin plus IVIG (2 g/kg) in the treatment of acute KD in children, and they found no benefits in high-dose aspirin compared to low-dose aspirin.[23] Hsieh et al. found that treatment with IVIG alone without aspirin in the acute stage of KD did not affect the response rate of IVIG treatment, duration of fever, or rate of CAL.[12] A meta-analysis by Durongpisitkul et al. showed that high-dose or lower-dose aspirin has a similar incidence of CAL in KD.[22] These findings are consistent with the current study in that high-dose aspirin treatment was not associated with any benefits in IVIG resistance, CAL formation, and duration of hospitalization.

Higher inflammatory markers and IVIG resistance may be associated with the occurrence of CAL in KD.[24] The level of CRP is elevated in KD patients, which then decreases after IVIG treatment.[2] In our patients, we found that patients who received high-dose aspirin had persistently higher CRP levels which did not significantly decrease despite IVIG therapy. Many studies have demonstrated that CRP level and fractional changes in CRP level may be a useful and important marker to predict initial IVIG resistance in KD patients.[25–27] Moreover, KD is an acute systematic vasculitis which causes hydrops of the gallbladder,[28] and we also previously showed that a higher CRP level is associated with sonographic gallbladder abnormalities[29]. Using a murine model of KD, it was found that IVIG, but not salicylate, effectively reduced the immune response leading to TNF-α expression.[30] Unexpectedly, pharmacological doses of salicylate were incapable of inhibiting TNF-α production but even enhanced its production.[30] This provides a hint that the usefulness of aspirin may be limited to its anticoagulant and antipyretic actions, which can be achieved at much lower doses.

It has been established that gastrointestinal bleeding and anemia are associated with the use of aspirin.[31] In addition, other major side effects of aspirin including hepatic toxicity,[32] sensorineural hearing loss[33] and Reye syndrome[34] may induce morbidity and mortality. In the current study, there was a significantly lower hemoglobin level in the high-dose aspirin group. We previously found that anemia in KD patients is related to a significantly increased hepcidin level that results in functional iron deficiency.[15] As is commonly known, hepcidin orchestrates both iron metabolism and the pathogenesis of anemia of inflammation. Higher hepcidin levels result in lower serum levels of iron and reduces the availability of iron for erythropoiesis. Here, we observed that high-dose aspirin influenced the decrease in hepcidin level after IVIG treatment. Thus, a higher hepcidin level may impair iron metabolism and decrease hemoglobin levels in KD patients receiving high-dose aspirin treatment. However, we do not exactly know how high-dose aspirin suppresses the decrease in hepcidin levels after IVIG and thus still needs further investigation. In this era of IVIG treatment, high-dose aspirin provides few benefits in the treatment of KD in the acute phase, and also actually induces lower hemoglobin levels and impairs the decrease in CRP and hepcidin levels after IVIG therapy.

## Conclusion

The results of the current study provide evidence that high-dose aspirin treatment in the acute phase of KD may impair the improvement of the inflammatory markers after IVIG therapy, but does not affect the treatment results. High-dose aspirin also caused a lower hemoglobin level that was associated with a higher hepcidin level after IVIG treatment. We suggest that it is unnecessary that children receive high-dose aspirin therapy in the acute phase of KD as no appreciable benefits were found in preventing the failure of IVIG therapy, CAL formation, or even shortening the duration of fever.

## Abbreviations

CAL: coronary artery lesions
CRP: C-reactive protein
ELISA: enzyme-linked immunoassay
G6PD: glucose-6-phosphate dehydrogenase
IVIG: intravenous immunoglobulin
KD: Kawasaki disease
